# Updating the Japanese Healthcare System to Meet the Needs of an Aging Society

**DOI:** 10.31662/jmaj.2024-0116

**Published:** 2024-10-03

**Authors:** Shotaro Kinoshita, Taishiro Kishimoto

**Affiliations:** 1 Hills Joint Research Laboratory for Future Preventive Medicine and Wellness, Keio University School of Medicine, Tokyo, Japan; 2 Graduate School of Interdisciplinary Information Studies, The University of Tokyo, Tokyo, Japan

**Keywords:** Aging society, Healthcare system, Health policy, Dementia, Japan

## To the Editors,

We read with great interest the opinion by Kanada et al. on Japan’s declining birth rate and aging population ^(1)^. In light of this article, there are a few additional points that we would like to discuss. In Japan, where geographical and linguistic barriers limit immigration, it may be difficult to avoid a declining birth rate and aging population. As medical professionals, we must discuss how to maintain and develop Japan’s medical care system in the face of an extremely low birth rate and aging population.

In Japan, the demand for inpatient care is expected to peak by 2040 ^(2)^. Consequently, it is estimated that medical and welfare workers, currently the third largest industry in Japan, will become the largest industry by 2035-2040 ^(2)^. Healthcare expenditures in 2040 are projected to increase to approximately ¥89 trillion, more than 1.6 times the 2023 level, resulting in a projected ¥27 trillion shortfall in financial resources if current tax rates remain unchanged ^(2)^. To maintain the current level of medical care, the government may need to reduce the supply of low-value care, promote task shifting from physicians to other professions, and improve efficiency through digital transformation.

The proper allocation of medical resources is an urgent issue. In Japan, although there has been an increase in the number of medical school students and revisions to the medical specialist system, the uneven distribution of medical resources remains unresolved, with shortages of obstetricians, pediatricians, and general surgeons, while the number of cosmetic surgeons has increased ^(2)^. There is also a shortage of human resources in university hospitals, and doctors at university hospitals are unable to devote time to research, leading to a decline in Japan’s research capabilities ^(3)^. In addition to promoting the consolidation of medical institutions, there is a need to establish enforceable policies for allocating medical resources. The government should also encourage the use of telemedicine to eliminate the disadvantages of the current uneven distribution of medical care for patients living in remote islands, remote areas, and the elderly, who have a heavy burden of making hospital visits ^(4)^.

It is also important to address the increasing number of patients with dementia. Japan’s Basic Law on Dementia calls for “symbiosis” with dementia ^(5)^. In addition to research on treatment and prevention of dementia, we as medical professionals should be actively involved in the development of services and products that enable patients with dementia to live in harmony with the society ^(5)^.

## Article Information

### Conflicts of Interest

S.K. declares no competing interests.

T.K. has received grants from Sumitomo Pharma and Otsuka Pharma; royalties or licenses from Sumitomo Pharma and FRONTEO; consulting fees from TechDoctor and FRONTEO; speaker’s honoraria from Sumitomo Pharma, Boehringer Ingelheim, Takeda, Astellas, Meiji Seika, and Janssen; and stocks from i2medical and TechDoctor.

### Author Contributions

S.K.: Conceptualization, writing ―original draft. T.K.: Writing ―review & editing.
